# Pathways to Wellbeing: Differentiated Associations Across Three Forms of Campus Engagement in a Diverse UAE University Context

**DOI:** 10.3390/ejihpe16080116

**Published:** 2026-08-12

**Authors:** Christopher Bryan, Ruba Ahmed, Tatiana Tairi, Oliver Bones, Muna Amr, Sariah Daouk

**Affiliations:** 1 Department of Psychology, American University of Sharjah, University City, Sharjah 26666, United Arab Emirates; g00094070@alumni.aus.edu (R.A.); ttairi@aus.edu (T.T.); 2 School of Humanities, Social Sciences and Health, University of Wollongong in Dubai, Dubai Knowledge Park, Dubai 20183, United Arab Emirates; oliverbones@uowdubai.ac.ae (O.B.); munaamr@uowdubai.ac.ae (M.A.); 3 Department of Psychology, Maudsley Health—Al Amal Psychiatric Hospital, Dubai 11425, United Arab Emirates; sariah.daouk@ehs.gov.ae

**Keywords:** social belonging, loneliness, stress mindset, international students, equity, path model, psychological distress

## Abstract

University students in diverse higher education contexts face concurrent risks: flourishing often below optimal levels alongside elevated depression and anxiety, though these are often addressed through generic, undifferentiated wellbeing provision. In diverse settings such as United Arab Emirates (UAE) universities, where international students constitute the majority of many student bodies, how campus activities relate to social belonging is both a wellbeing question and an equity issue. This study examined how extracurricular participation, sport (a subset of extracurricular participation), and exercise were associated with student wellbeing among 801 students across two UAE institutions. Extracurricular participation was associated with social belonging, which showed significant indirect associations with flourishing, depression, and anxiety, consistent across covariate adjustment and robustness checks. Exercise showed a similarly consistent direct association with flourishing; it was also associated with lower depression and anxiety in the primary model, though these associations were less robust than the flourishing finding and did not hold under further sensitivity checks. Sport showed a small association with stress mindset and anxiety, though this relies on a measure with low reliability and should be treated as tentative. Loneliness was not significantly associated with any engagement form. These findings point to a differentiated picture of campus engagement, in which extracurricular participation and exercise show the clearest links to wellbeing, while sport’s association with stress appraisal requires further evidence.

## 1. Introduction

### 1.1. Pathways to Wellbeing: Differentiated Associations Across Three Forms of Campus Engagement in a Diverse UAE University Context

Progression to University is one of the most significant life transitions a young adult undergoes, involving not only increased academic demands but the disruption of established social networks and the challenge of constructing a social identity within an unfamiliar institution (Veldman et al., 2019). For international students (i.e., those studying outside their country of origin) these challenges are compounded by cultural adjustment and the absence of community networks built over years (Berry, 2005). While international students represent a growing but structurally vulnerable minority in most national university systems, the United Arab Emirates (UAE) presents a markedly different landscape: non-national students constitute the majority of newly enrolled students at over half of UAE higher education institutions (Ministry of Higher Education and Scientific Research, 2025). In this context, challenges typically associated with minority status become a majority experience for the student body, creating a distinctive demographic configuration that warrants further scholarly investigation. The question of how universities support social integration and psychological wellbeing is not a peripheral concern but a central institutional responsibility, and one with direct implications for equity in educational participation. Despite the scale of this challenge, Oduwaye et al. (2023) systematic review of 175 studies on international student challenges published over 21 years found that the UAE accounted for approximately 1% of the research, despite hosting student populations whose diversity and international composition rival those of the most-studied Anglo-American contexts, and that the challenges facing international students showed no meaningful improvement across the entire period reviewed. The dominant literature continues to reflect Western, primarily North American and European, student populations, and the present study responds directly to that gap.

### 1.2. Social Belonging as the Central Wellbeing Resource

Among the psychological resources most relevant to student wellbeing, social belonging occupies a particular position. Belongingness is the subjective sense of being a valued member of a social group, reflecting the fundamental human need for stable and meaningful social bonds (Baumeister & Leary, 1995), and in educational settings, it is consistently linked to lower depression and anxiety levels, higher academic engagement, and greater flourishing (Allen et al., 2021; Gopalan et al., 2022).

For students entering new social environments, the experience Walton and Cohen (2007) term belonging uncertainty, persistent doubt about whether one is accepted and valued within an institution, can undermine wellbeing even in the absence of overt exclusion, and is especially pronounced among those navigating unfamiliar cultural, linguistic, or institutional norms (Walton & Cohen, 2011). In a setting such as the UAE where the majority of students have relocated from different cultural backgrounds and face some degree of cultural transition, belonging uncertainty is likely widespread among the student body rather than a marginal experience affecting only small, marginalised groups.

This vulnerability can be understood through Hobfoll’s (1989) Conservation of Resources framework, which proposes that stress arises from the loss or threat of valued social and psychological resources. For students entering an unfamiliar institutional environment, the resources most central to wellbeing are precisely those most disrupted: established social networks, cultural familiarity, and the identity continuity that protects self-concept (i.e., the perceived coherence between one’s prior and current social roles) (Berry, 2005; R. A. Smith & Khawaja, 2011; Yeh & Inose, 2003). Loneliness, on this account, is not merely a feeling but a signal of resource loss, and campus activities that build new social resources, particularly belonging, represent one of the most accessible institutional responses to this structural vulnerability. In the UAE, students may face additional ongoing challenges in developing stable friendships with diverse cultural and linguistic groups, where social interaction does not translate directly into a sense of belonging within the university student body.

Loneliness is the subjective sense of social deprivation; it is both a symptom of threatened social resources and a risk factor for further deterioration across wellbeing dimensions (Hawkley & Cacioppo, 2010). Although belonging and loneliness are related, they are empirically distinct: belonging reflects positive appraisal of social membership, while loneliness reflects felt social insufficiency. Examining both allows the present study to assess wellbeing from complementary angles. These constructs operate across both dimensions of what Keyes (2002) describes as a dual continua model of mental health. Flourishing reflects optimal positive functioning characterised by purpose, engagement, and positive affect, while psychological distress captures clinical symptom burden through conditions such as depression and anxiety. Although related, the two dimensions are empirically separable, meaning that reducing symptoms does not automatically generate flourishing and that treating them as equivalent outcomes obscures how different campus activities affect each. This distinction is consistent with clinical and psychiatric traditions, which hold that the absence of mental disorder does not equate to positive mental health (Engel, 1977), and with dual-continua psychological research that conceptualise and measure psychological distress separately from wellbeing (Diener et al., 2010). A university system focused only on reducing depression and anxiety, therefore, neglects students who are not clinically unwell but still failing to thrive. The present study examines flourishing and psychological distress as distinct outcomes.

### 1.3. Three Forms of Campus Engagement, Differentiated Associations with Wellbeing

Structured campus activities create smaller social communities within the broader institution where students can form peer relationships and develop a sense of institutional identity (Tinto, 1993). Extracurricular participation has been linked to university belonging specifically among international students (Thies & Falk, 2023), and a recent path model at a comparable UAE institution found that campus environment and relationship quality was associated with belonging, which in turn shaped satisfaction with the university experience (Kassab et al., 2024). Undergraduate students meeting recommended physical activity guidelines report fewer depressive symptoms than insufficiently active peers (El Ansari & Salam, 2021), university sport participation is associated with better health-related quality of life (Snedden et al., 2019), and extracurricular involvement has been linked to broader student development (Foubert & Urbanski, 2006). These findings collectively support the value of campus engagement, but each has examined one form in isolation; no prior study has tested whether extracurricular participation, sport, and exercise operate through differing mechanisms when examined simultaneously within the same analytical framework.

Most studies further operationalise participation as a single binary variable without distinguishing the structurally and psychologically distinct activities it encompasses (Dearth-Wesley et al., 2026). The present study addresses both gaps, examining three forms of engagement separately, each associated with a different theoretical mechanism.

Extracurricular participation, involvement in clubs, cultural societies, volunteer programmes, and interest-based organisations, primarily requires no physical skill or competitive confidence and creates environments organised around shared identity. Social Identity Theory (Tajfel & Turner, 1979) suggests such group membership generates not merely social contact but an identity that buffers against stress and builds institutional belonging.

Sport participation (a subset of extracurricular participation rather than a fully independent category, as measured here) involves repeated exposure to challenge, pressure, and performance evaluation, which may cultivate a more adaptive stress mindset, the belief that stress can be manageable and even beneficial (Crum et al., 2013). Self-Determination Theory suggests sport environments supporting competence and relatedness enhance wellbeing (Deci & Ryan, 2000), though performance-oriented climates can carry psychological costs for some athletes (Reinboth & Duda, 2006).

Exercise, self-directed activity outside any formal sport structure, requires no institutional affiliation, competitive skill, or existing social network, and its benefits for depression and anxiety are among the most consistently replicated findings in health psychology (Liu et al., 2025). Because it remains accessible to students facing linguistic, cultural, or social barriers to institutional participation, exercise may offer a route to wellbeing promotion less dependent on organised participation.

Together, these three forms of engagement are theorised to operate through different mechanisms: identity-based belonging for extracurricular activities, adaptive stress appraisal for sport, and direct symptom reduction for exercise. Because these mechanisms are conceptually distinct, the forms of engagement are not necessarily mutually exclusive but potentially complementary, though the strength of evidence for each mechanism, examined below, differs considerably.

### 1.4. The Present Study

The present study examines whether extracurricular participation, sport, and exercise are differentially associated with wellbeing, operating through different psychological correlates and showing different patterns of association with outcomes across Keyes’ dual continua. Analyses proceeded in two stages: group-level comparisons examining baseline differences between participants and non-participants, and a path model simultaneously testing whether differing mediating mechanisms, specifically social belonging, loneliness, and stress mindset, explain why each engagement form predicts the outcomes it does. Two hypotheses are proposed.

**Hypothesis 1.**  *Predicts that students engaged in extracurricular activities, sport, or regular exercise will report significantly better psychological outcomes than non-participating peers across both continua, and that each engagement type will show a different profile: extracurricular activities more strongly associated with social belonging, sport with stress mindset, and exercise with the broadest symptom-level outcomes.*

**Hypothesis 2.**  *Predicts that the three engagement forms will show differing patterns of association with the psychological mediators tested, with extracurricular participation most strongly associated with social belonging, sport with stress-appraisal processes, and exercise with direct associations with flourishing, depression, and anxiety.*

## 2. Method

### 2.1. Participants and Procedure

Data were collected during the 2024–2025 academic year from undergraduate and postgraduate students enrolled at two English-medium universities in the United Arab Emirates. Both institutions enrol predominantly international student populations across a wide range of nationalities, reflecting the broader character of higher education in the UAE. Ethical approval was granted by the Institutional Review Board (reference: 24-080, approved 23 May 2024), and participation was voluntary and anonymous throughout. Informed consent was obtained at the start of the survey prior to any data collection. Recruitment used campus flyers, faculty referrals during class time, and a university-wide email. At the end of the survey, participants were directed to an optional external link to enter a prize draw for one of five AED 350 vouchers. The survey instrument was the Healthy Minds Study (Healthy Minds Network, n.d.), shared with the authors by Daniel Eisenberg for independent administration following the same survey design and approach.

The final sample comprised 801 students who completed at least the demographic and outcome sections of the survey; missing data on individual variables were retained and addressed analytically rather than through case-wise exclusion (see Section 2.3). The sample was predominantly female (66.1%, *n* = 529; male: 33.9%, *n* = 271), with ages ranging from 18 to 49 years (*M* = 20.70, *SD* = 3.64). The majority were enrolled in Bachelor’s degree programmes (90.0%), with smaller proportions at Master’s (8.3%) and doctoral (1.1%) level. The sample was highly multinational, with over 50 nationalities represented; the largest groups were Indian (26.0%), Emirati (12.5%), Egyptian (10.1%), and Pakistani (8.5%). Fields of study included Engineering and Technology (33.9%), Business and Management (23.3%), and Social Sciences (11.4%), among others. Consistent with the diversity of UAE higher education, 43.4% of participants (*n* = 348) were born in the United Arab Emirates and 56.6% (*n* = 453) were born elsewhere; a formal international student status variable distinguishing citizenship from residency or visa status was not collected.

With respect to campus engagement, 55.2% (*n* = 439 of 795 valid responses) reported extracurricular involvement, 11.4% (*n* = 91 of 801) reported organised sport participation, and 64.1% (*n* = 472 of 736 valid responses) met the active exercise threshold of one or more hours of moderate-to-vigorous physical activity per week. Percentages are calculated using the valid responses for each variable given variable-specific missingness (Supplementary Table S11). Participation rates in extracurricular activities, sport, and exercise stratified by gender and degree level are reported in Supplementary Tables S1 and S2.

### 2.2. Measures

#### 2.2.1. Flourishing

Flourishing was assessed using the Flourishing Scale (Diener et al., 2010), an 8-item measure of eudaimonic wellbeing covering purpose, optimism, and social relationships, rated on a 7-point scale (1 = strongly disagree to 7 = strongly agree; total scores range 8–56; scores ≥ 48 indicate high flourishing). Internal consistency in the present sample: α = 0.87, ω = 0.871.

#### 2.2.2. Depression

Depression was measured using the Patient Health Questionnaire-9 (PHQ-9; Kroenke et al., 2001), a 9-item screening instrument assessing depressive symptom frequency over the previous two weeks. Items were scored on a four-point Likert-scale ranging from 0 (Not at all) to 3 (Nearly every day). Total scores range from 0 to 27. Internal consistency: α = 0.86, ω = 0.860.

#### 2.2.3. Anxiety

Anxiety was measured using the Generalised Anxiety Disorder-7 scale (GAD-7; Spitzer et al., 2006), a 7-item instrument assessing anxiety symptom frequency rated on a four-point Likert-scale ranging from 0 (Not at all) to 3 (Nearly every day). Total scores range from 0 to 21. Internal consistency: α = 0.91, ω = 0.914.

#### 2.2.4. Social Belonging 

Social Belonging was assessed using a 4-item composite drawn from an adapted version of the Sense of Social and Academic Fit Scale (Walton & Cohen, 2007) and the Perceived Cohesion Scale (Bollen & Hoyle, 1990), both adapted within the Healthy Minds Study instrument. Items were rated on a 5-point Likert-scale, ranging from 1 (strongly agree) to 5 (strongly disagree); positively worded items were reverse-scored so that higher composites indicate greater belonging. This composite has been validated in large-scale undergraduate samples: Dearth-Wesley et al. (2026) report α = 0.78 in *N* > 16,000 US undergraduates using the identical 4-item measure. Internal consistency in the present sample: α = 0.75, ω = 0.761.

#### 2.2.5. Loneliness

Loneliness was assessed using the 3-item UCLA Loneliness Scale (UCLA-LS3; Russell, 1996), measuring subjective social isolation, lack of companionship, and feeling left out. Items were summed (range 3–9; higher = greater loneliness). Internal consistency: α = 0.84, ω = 0.844.

#### 2.2.6. Stress Mindset 

Stress Mindset was measured using an abbreviated 4-item version of the Stress Mindset Measure (SMM; Crum et al., 2013). Two negatively worded items were reverse-scored; all four items were summed (range 4–20; higher = stronger stress-is-enhancing orientation). Internal consistency was α = 0.40, ω = 0.544, reflecting the known partial independence of the SMM’s positive and negative subscales rather than random measurement error (Crum et al., 2013). A confirmatory factor analysis testing single- and two-factor solutions supported the two-factor structure: the positive and negative subscales were statistically independent (factor correlation = −0.001), and one reversed item showed a negative corrected item-total correlation, indicating it does not cohere with the summed score in the expected direction (Supplementary Table S3). Given this, all findings involving stress mindset are treated as exploratory throughout this paper.

#### 2.2.7. Extracurricular Participation 

Extracurricular Participation was assessed with the item “How much time do you spend during a typical week participating in campus activities, organizations, sports, or extracurriculars connected to University?” (1 = Less than 1 h/week to 7 = More than 20 h/week). Students reporting any participation (response ≥ 2) were coded as participating (1); others as non-participating (0).

#### 2.2.8. Sport Participation 

Sport Participation was assessed with the item “How much time do you spend participating in your sport (including practice, team meetings, workouts, etc.)?” using the same 7-point scale. Because this item and the extracurricular item overlap conceptually, sport participation was structurally nested within extracurricular participation: no student reported sport involvement without also reporting extracurricular involvement (Supplementary Table S4). The simultaneous path model therefore isolates sport’s association net of general extracurricular engagement. Students reporting any sport involvement (response ≥ 2) were coded as participating (1); others as non-participating (0).

#### 2.2.9. Exercise Participation 

Exercise Participation was assessed with the item “In the past 30 days, about how many hours per week on average did you spend exercising? (Include any exercise of moderate or higher intensity…)” on a 5-point scale (1 = Less than 1 h, 2 = 1–2 h, 3 = 2–3 h, 4 = 3–4 h, 5 = 5 or more hours). Students selecting option 1 were coded as low exercise (0); all others as active exercise (1; *n* = 472, 64.1%). This threshold distinguishes students reporting less than 1 h of weekly exercise from those reporting at least 1 h; it does not indicate sedentary behaviour specifically, as sedentary behaviour was not directly measured. The distinction is motivated by literature suggesting that the largest mental health gains from physical activity may occur in the transition from very low reported activity to any regular activity, rather than from further increases at higher levels (Chekroud et al., 2018). This threshold should not be read as representing compliance with, or departure from, established physical activity guidelines such as the WHO’s 150 min/week recommendation. A sensitivity analysis using a higher threshold (≥2–3 h/week) is reported in Supplementary Table S5.

Formal psychometric validation of these instruments in UAE or other culturally diverse international samples has not been established; while the sample was highly multinational, all measures were administered in English, and cultural or linguistic variation in item interpretation cannot be ruled out.

### 2.3. Data Analysis

All analyses were conducted in R (Version 4.2.1; R Core Team, 2022) using the lavaan package for path modelling (Version 0.6-12; Rosseel, 2012), the psych package for reliability analysis (Revelle, 2024), and the effectsize package for effect size computation (Ben-Shachar et al., 2020). Preliminary data preparation was conducted in jamovi (Version 2.7; The jamovi project, 2025).

Group difference analyses used Welch’s independent-samples *t*-test separately for each engagement predictor across all six outcome and mediator variables. Welch’s correction was applied throughout to account for unequal group sizes inherent to binary participation variables (Delacre et al., 2017). Cohen’s *d* is reported as the effect size measure, with benchmarks of 0.20, 0.50, and 0.80 interpreted as small, medium, and large, respectively (Cohen, 1988). Given 18 comparisons across engagement types and outcomes, Benjamini–Hochberg false discovery rate (FDR) correction was applied (Benjamini & Hochberg, 1995; Supplementary Table S6), with corrected values taking interpretive precedence.

A path model was estimated using maximum likelihood estimation, specifying all three engagement types as exogenous predictors, social belonging, loneliness, and stress mindset as mediators, and flourishing, depression, and anxiety as outcomes. Residual covariances among the three outcomes and among the three mediators were freely estimated. Sport, exercise, and extracurricular participation variables were recoded to binary indicators (0 = no participation, 1 = participation) prior to modelling. Following recommended practice (Kline, 2016), a saturated model (df = 0) served as a specification check before a trimmed model was estimated retaining paths that were statistically significant (*p* < 0.05) or theoretically essential regardless of significance (MacCallum et al., 1992). The trimmed model was formally compared to the saturated model via a chi-square difference test; a non-significant Δχ^2^ indicates the imposed constraints did not significantly worsen fit, justifying parsimony. Model fit was evaluated using CFI, TLI, RMSEA, and SRMR (Hu & Bentler, 1999). Covariances among the three binary participation predictor variables were freely estimated in all models, consistent with lavaan’s default treatment of exogenous variables (Rosseel, 2012); estimated values are reported in Supplementary Table S7.

Sensitivity analyses addressed unmeasured confounding (covariate-adjusted model: gender, age, institution), distributional violations (MLR estimation), and missing-data assumptions (multiple imputation, *m* = 20); full model specifications, R^2^, and predictor covariances are reported in Supplementary Tables S7–S10 and Supplementary Code.

Missing data on outcome and mediator variables were handled using full information maximum likelihood (FIML; Graham, 2009). Variable-level missingness ranged from 0% to 16.0% (Supplementary Table S11). Participants excluded from the path model due to missing predictor data (*n* = 68) reported significantly lower depression scores than included participants (Supplementary Table S12), suggesting the missing-at-random assumption may not hold perfectly with respect to depression; a multiple imputation sensitivity analysis (Supplementary Table S10) confirmed the primary findings were robust to this concern. Of 801 participants, 733 provided complete data on all three engagement predictors and contributed to the path model; 68 cases with missing predictor values were excluded aligning with lavaan’s default handling of exogenous variables (Rosseel, 2012). Indirect effects were estimated using non-parametric bootstrapping with 5000 resamples; bias-corrected accelerated (BCa) 95% confidence intervals are reported, with indirect effects considered significant when the interval excludes zero (Preacher & Hayes, 2008).

## 3. Results

### 3.1. Preliminary Analyses

Descriptive statistics and bivariate correlations are presented in Table 1. Across the full sample, mean flourishing scores (*M* = 41.08, *SD* = 8.61) fell below the established high-flourishing threshold of 48, and mean depression (*M* = 11.94, *SD* = 7.14) and anxiety (*M* = 10.24, *SD* = 6.04) scores were in the mild-to-moderate range on established severity classifications; 59.1% of participants scored above the PHQ-9 moderate-depression threshold (≥10) and 50.6% scored above the GAD-7 moderate-anxiety threshold (≥10) (Supplementary Table S13), consistent with prior evidence of elevated symptom burden in university populations (Stallman, 2010). As both instruments are screening tools rather than diagnostic measures, these proportions indicate elevated symptom risk rather than confirmed clinical diagnosis. Social belonging was positively associated with flourishing (*r* = 0.48) and negatively associated with depression (*r* = −0.35) and anxiety (*r* = −0.28), while loneliness showed the inverse pattern across all three outcomes. Stress mindset was modestly but significantly associated with flourishing (*r* = 0.14) and negatively associated with depression (*r* = −0.13) and anxiety (*r* = −0.17). The moderate negative correlation between belonging and loneliness (*r* = −0.48) confirmed that, while related, the two constructs captured distinct aspects of social experience. These levels are broadly aligned with cross-national evidence of elevated depression and anxiety among university students in Arab contexts (Kronfol et al., 2018), situating the present sample within a wider pattern of psychological distress in Gulf higher education.

### 3.2. Group Difference Profiles

Group comparisons are presented in Table 2. Hypothesis 1 predicted that each engagement form would be associated with better psychological outcomes than non-participation and that the three would show a different profile consistent with their theoretical mechanisms. All three forms were associated with significantly higher flourishing, providing broad support for the first part of this prediction. The profiles were otherwise different. Extracurricular participation showed a strong and selective social effect: participants reported significantly greater social belonging than non-participants, with the largest effect size observed across any comparison in the study (*d* = 0.65), alongside higher flourishing and a modest reduction in depression that did not survive false discovery rate correction (Supplementary Table S6). Extracurricular participants also reported significantly higher stress mindset scores in this bivariate comparison (*d* = 0.16, *p* = 0.037), though this difference likewise did not survive FDR correction and did not emerge as a significant path once all three engagement types were modelled simultaneously (see Path Model below). No significant differences emerged for anxiety or loneliness. Sport participants reported significantly higher flourishing, greater belonging, and higher stress mindset scores than non-participants, with uniformly small effect sizes, but no significant differences for depression, anxiety, or loneliness. Exercise showed the broadest clinical reach: active students reported significantly lower depression and anxiety, lower loneliness, and higher flourishing than low-exercise peers, with no association with social belonging or stress mindset. This pattern of differentiated profiles broadly supports Hypothesis 1 and motivated the path model analyses that follow.

After FDR correction across all 18 comparisons, three effects that were significant at the conventional threshold, extracurricular participation differences in stress mindset and depression, and sport participation differences in social belonging, no longer reached significance (Supplementary Table S6) and are interpreted as descriptive rather than confirmed group differences.

### 3.3. Path Model

**Figure 1:**
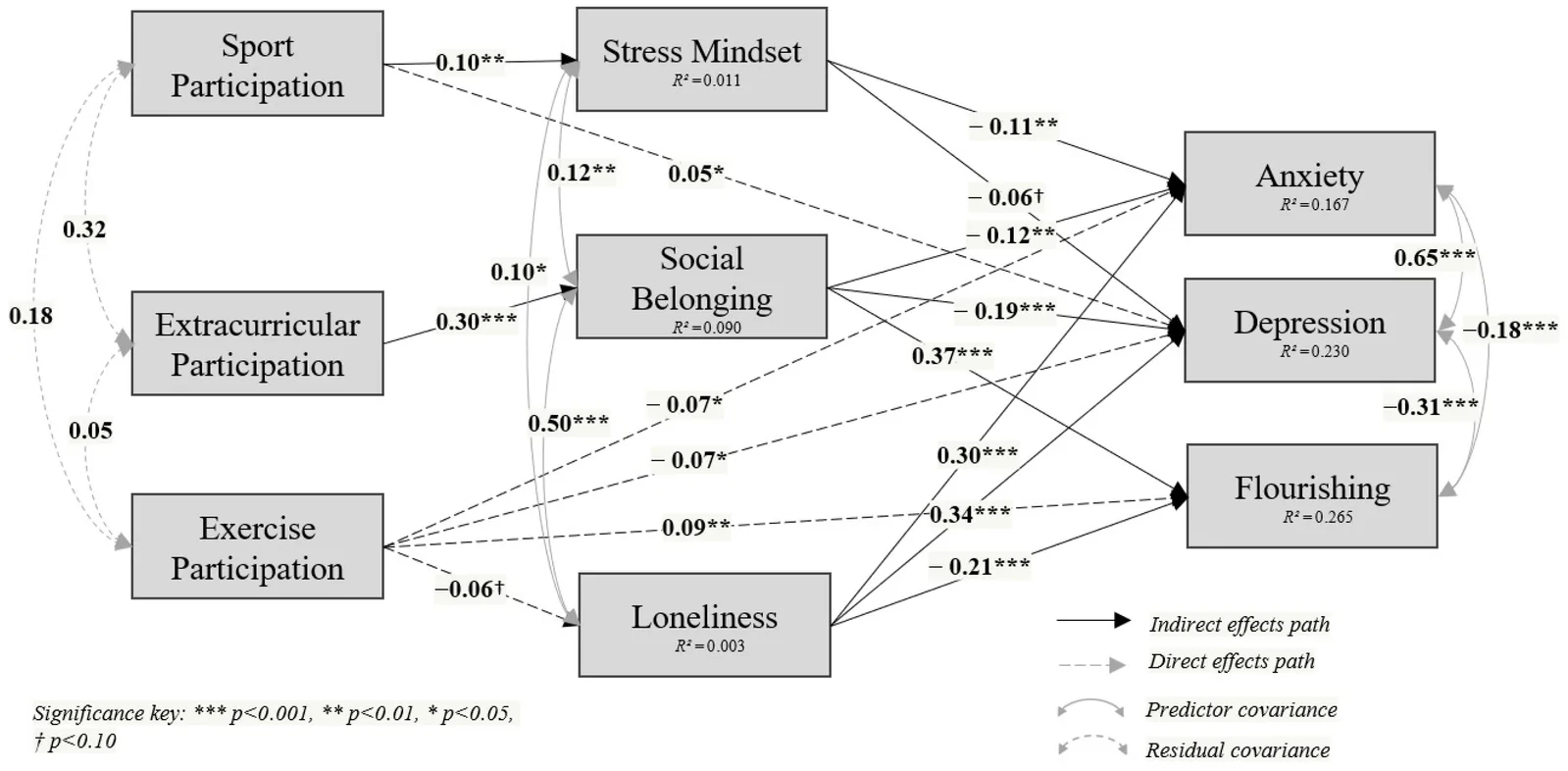
Trimmed path model with standardised coefficients (*N* = 733). Notes. Mediator boxes shaded. Model fit: χ^2^(12) = 20.08, *p* = 0.066; CFI = 0.994; TLI = 0.983; RMSEA = 0.030 [0.000, 0.053]; SRMR = 0.020. Bootstrapped indirect effects reported in Table 3. Coefficients represent cross-sectional associations and should not be interpreted as evidence of directional or causal effects.

The trimmed model showed good fit to the data: χ^2^(12) = 20.08, *p* = 0.066; CFI = 0.994; TLI = 0.983; RMSEA = 0.030, 90% CI [0.000, 0.053]; SRMR = 0.020, and did not significantly worsen fit relative to the saturated model, Δχ^2^(12) = 20.08, *p* = 0.066. As paths were retained based on their significance in this same sample, these fit indices reflect model specification rather than independent confirmatory validation; the full saturated model is reported in Supplementary Table S7. The full model, including all retained paths, residual and predictor covariances, and R^2^ for each mediator and outcome, is shown in Figure 1; read left to right, the diagram traces the hypothesised sequence from campus engagement to psychological mediator to wellbeing outcome. Full parameter estimates, standardised coefficients, and bootstrapped confidence intervals are presented in Table 3.

#### 3.3.1. Pathways from Campus Engagement to Psychological Mediators (A-Paths)

Extracurricular participation was uniquely associated with social belonging (β = 0.30, *p* < 0.001), with neither sport nor exercise contributing unique variance to belonging once all three predictors were included. Sport participation was uniquely associated with stress mindset (β = 0.10, *p* = 0.007). Exercise showed a small negative association with loneliness (β = −0.06, *p* = 0.090), but this association was not statistically significant and its bootstrapped confidence interval included zero. This pattern offers partial support for the functional specificity predicted by Hypothesis 2: extracurricular participation was uniquely associated with belonging and sport was uniquely associated with stress mindset, whereas no engagement form showed a reliable association with loneliness.

When gender, age, and institution were added as covariates in a sensitivity model, all three A-paths retained their sign and significance pattern (Supplementary Table S8), as did the majority of B-paths and indirect effects. However, the exercise associations with depression and anxiety attenuated to non-significance after covariate adjustment, coinciding with a substantially higher rate of exercise participation among men (74.2%) than women (58.8%) and significantly higher depression (β = 0.133, *p* < 0.001) and anxiety (β = 0.201, *p* < 0.001) among women independent of exercise. This indicates that part of exercise’s unadjusted association with lower depression and anxiety reflects gender composition rather than an independent effect of exercise itself.

Findings were robust to MLR estimation and multiple imputation (Supplementary Tables S9 and S10); the exercise associations with depression and anxiety weakened further under a higher activity threshold, while extracurricular and sport paths were unaffected (Supplementary Table S5).

#### 3.3.2. Pathways from Psychological Mediators to Wellbeing Outcomes (B-Paths)

Social belonging and loneliness were the strongest mediating variables across both continua. Belonging was associated with higher flourishing (β = 0.37, *p* < 0.001) and lower depression (β = −0.19, *p* < 0.001) and anxiety (β = −0.12, *p* = 0.004). Loneliness showed the complementary pattern, associated with lower flourishing (β = −0.21, *p* < 0.001) and higher depression (β = 0.34, *p* < 0.001) and anxiety (β = 0.30, *p* < 0.001). Stress mindset was associated with lower anxiety (β = −0.11, *p* = 0.002) and showed a marginal negative association with depression (β = −0.06, *p* = 0.078), but was not significantly associated with flourishing

#### 3.3.3. Direct Effects

Exercise showed a significant direct association with flourishing after accounting for its indirect pathway through loneliness (β = 0.09, *p* = 0.006), and this association remained robust to covariate adjustment and to a higher activity threshold. Exercise was also associated with lower depression and anxiety in this unadjusted primary model (both β = −0.07, *p* < 0.05); however, these two associations were not robust, attenuating to non-significance under covariate adjustment and weakening further at a higher activity threshold. Sport showed a small but significant positive direct path to depression (β = 0.05, *p* = 0.036), a suppression effect whereby, once stress mindset and belonging are controlled, sport carries a residual positive association with depressive symptoms.

#### 3.3.4. Evaluation of Hypothesis 2

Hypothesis 2 received differentiated support across its three components. Extracurricular participation showed significant indirect associations with flourishing, depression, and anxiety through belonging, with all three 95% bootstrapped confidence intervals excluding zero. Sport showed a significant indirect association with anxiety through stress mindset, though the corresponding association with depression was non-significant under bootstrapping; given the stress mindset measure’s poor internal consistency and its statistically independent item subscales (Supplementary Table S3), this pathway is reported as a data pattern awaiting confirmation with a more reliable measure, not as an established indirect effect. Exercise showed a significant direct association with flourishing that was robust to covariate adjustment and to a higher activity threshold. It was also associated with lower depression and anxiety in the unadjusted primary model, but these associations were not robust, attenuating under covariate adjustment and weakening further at a higher activity threshold. Its hypothesised indirect association through loneliness was non-significant under bootstrapping. Overall, exercise’s association with wellbeing in this sample was most consistent for flourishing.

## 4. Discussion

The present study examined how three overlapping forms of campus engagement, extracurricular participation, sport, and exercise, were associated with student wellbeing across Keyes’ dual continua. The findings show a differentiated rather than uniform pattern: associations were largely specific to particular engagement forms and mediators rather than consistent across all three. All data were collected at a single time point, and the associations reported below cannot establish direction of influence; reverse or reciprocal pathways, for example, students who already feel they belong or are already flourishing being more likely to engage in campus activities, are equally plausible, and longitudinal or experimental designs are needed to test directionality. The most consequential association, and the one most directly relevant to the design of inclusive university environments, concerns social belonging.

### 4.1. Social Belonging as the Strongest Correlate of Wellbeing

Extracurricular participation was the only engagement form significantly associated with social belonging, which in turn showed significant indirect associations with flourishing, depression, and anxiety. This pattern is aligned with the identity-based account of belonging development proposed by Social Identity Theory (Tajfel & Turner, 1979): when group membership is grounded in shared cultural identity or interest rather than physical proximity or competition, it is more likely to produce the stable felt sense of social membership that constitutes genuine belonging. In a campus environment where over 50 nationalities share the same institution, this mechanism may carry particular force. Cultural societies, language groups, and interest-based clubs offer students the experience of being understood in terms of their background and perspective, not merely encountered in close physical proximity. It is this quality of recognition that may distinguish their effect on belonging from the social contact generated by sport or exercise. The finding that sport and exercise were not significantly associated with belonging once examined simultaneously is consistent with this interpretation: social contact alone may not be associated with belonging to the same degree as environments offering identity-affirmation. This remains a plausible explanation rather than a demonstrated mechanism, since identity affirmation itself was not directly measured. One implication, warranting longitudinal or intervention testing before adoption, is that programming organised around shared cultural identity may be more strongly associated with belonging than physical activity programmes; the present cross-sectional data indicate only that current extracurricular participation and current belonging co-occur more strongly than sport or exercise participation and belonging, not that such programming would causally increase it.

This interpretation is consistent with evidence from comparable Arab Gulf region contexts. Kassab et al. (2024) found that campus environment quality and student relationships were associated with belonging at a similarly diverse UAE institution, which was in turn associated with student satisfaction. Their study did not compare multiple forms of campus engagement against one another; the present findings extend this work by showing that engagement types differ in their association with belonging, with extracurricular participation significantly associated with belonging while sport and exercise are not, even when all three are tested simultaneously in the same model. This pattern is also broadly consistent with a positive bivariate correlation between belonging and flourishing reported by Dearth-Wesley et al. (2026) in a US sample of more than 16,000 undergraduates, suggesting this association may generalise across different national and cultural contexts, though this remains to be tested directly.

This matters especially in the first weeks of enrolment, when Walton and Cohen (2007) show belonging uncertainty is at its peak and when the social environments students enter most shape their subsequent trajectory. Active onboarding into extracurricular communities, visible cultural representation within clubs and societies, and institutional messaging that frames these activities in terms of belonging rather than achievement are all practical steps supported by the present evidence. The design of extracurricular activities matters as much as their provision: empirical evidence indicates that activities are most effective in improving belonging when they integrate social, academic, and informal belonging dimensions simultaneously, rather than addressing only one aspect of students’ campus experience (De Sisto et al., 2022). Walton and Cohen’s (2011) demonstration that even brief institutional signals about belonging can meaningfully improve student outcomes reinforces that these do not require large-scale investment to be effective.

### 4.2. Loneliness: A Powerful Predictor That Campus Activity Alone Does Not Resolve

Loneliness showed among the strongest associations with depression and anxiety in the model, yet none of the three engagement forms showed a significant adjusted association with it when examined simultaneously. This finding deserves careful attention. Research by Qualter et al. (2015) describes loneliness as a motivational state that can become self-reinforcing: students who feel socially deprived may disengage from the very social situations that could alleviate their isolation, anticipating rejection or misunderstanding. Participation in social activities might not translate directly into perceived integration or reduced loneliness. Structured participation programmes, which typically require a degree of social confidence to enter, may therefore be least accessible to the students most at risk of loneliness, particularly newly arrived international students who face acculturative stress and the absence of established social networks (R. A. Smith & Khawaja, 2011; Sümer et al., 2011).

The distinction between belonging and loneliness matters practically. A student who feels accepted within a specific club may still feel socially isolated across the rest of their university life. Reducing loneliness appears to require more than providing structures for social contact; it requires generating consistent, low-stakes social connection across multiple contexts. Peer-to-peer mentoring, structured academic group work that creates repeated informal contact, and residential arrangements that facilitate cross-cultural interaction have each shown promise in this regard (Shook & Clay, 2012), and universities in diverse contexts may need to invest in this alongside extracurricular provision rather than assuming that club participation will reach students who are already withdrawn.

### 4.3. Sport, Stress Appraisal, and the Limits of the Present Measure

The literature on sport and psychological adaptation suggests a plausible stress-appraisal mechanism worth testing; repeated exposure to structured challenge may, in principle, help individuals develop a more adaptive stress mindset, the belief that stress can be manageable and even beneficial (Crum et al., 2013), and sport participation has separately been associated with psychological resilience and coping capacity beyond what exercise alone provides (Eime et al., 2013). The present study cannot speak to this possibility with confidence. The Stress Mindset Measure showed poor internal consistency in this sample, and a confirmatory factor analysis indicated its positive and negative items form statistically independent subscales rather than a coherent construct (Supplementary Table S3); the summed score does not, on this evidence, provide a sufficiently coherent measure to support a firm interpretation of sport’s association with stress appraisal. The small association observed between sport and stress mindset, and its indirect association with anxiety, should therefore be read as a data pattern awaiting confirmation with a more psychometrically sound instrument, rather than as evidence establishing a distinct sport–stress-appraisal pathway. Notably, this pattern was present among men but not women (Supplementary Table S2), with women’s sport participation rate roughly half that of men’s (8.3% vs. 17.3%); this may bear on equitable programme design but cannot be evaluated further given the measurement limitations above.

The small positive direct association between sport participation and depression warrants caution: this path emerged only within the adjusted model, and the zero-order correlation between sport and depression was negligible, indicating the adjusted association likely reflects a suppression effect rather than a genuine cost of sport participation. We raise this as a pattern requiring replication, not as evidence that competitive sport increases depression risk. Kuettel and Larsen (2020) identified performance pressure, ego-threatening climates, and the experience of failure as risk factors for depression among athletes in organised sport settings. Research on motivational climate in sport consistently shows that environments emphasising personal improvement and effort are associated with better psychological outcomes than those emphasising competitive ranking and social comparison (Ames, 1992; Reinboth & Duda, 2006). This suggests, though it would require longitudinal testing to confirm, that university sport coordinators and coaches may shape psychological outcomes through the climate they create. Whether a mastery-oriented sport environment would strengthen the exploratory stress-appraisal pattern observed here cannot be determined from the present data. The same is true of its potential effect on the small positive association with depression that emerged in the adjusted model; both would require direct testing with a more reliable measure.

### 4.4. Equity and Institutional Design

The following four institutional strategies are presented as testable candidates informed by the present associations and the broader intervention literature, and none should be adopted as policy without prospective or longitudinal evaluation. Across the three engagement forms, the barriers to access differ in kind: extracurricular activities require social confidence and cultural familiarity; organised sport requires competitive skill and tolerance of ego-threatening environments; exercise requires overcoming psychological and sometimes cultural obstacles to physical self-presentation. The students who stand to gain most from campus engagement are often those facing the greatest barriers to it.

#### 4.4.1. Strategy 1: Structured Extracurricular Onboarding with Cultural Representation

Extracurricular participation was the strongest and only significant predictor of social belonging in the present study, consistent with prior evidence that such participation supports belonging and broader community development (De Sisto et al., 2022). Supporting this pathway in highly diverse student bodies requires intentional, systematic effort rather than passive provision. We recommend a campus-wide inclusive strategy ensuring students from diverse cultural backgrounds are represented at all stages of extracurricular planning, embedding mechanisms for accessibility, student voice, and agency. Concrete steps include: systematically analysing student demographic composition to inform activity design, given documented disparities in both belonging (Gopalan & Brady, 2020) and extracurricular access (Kaufman & Gabler, 2004) across demographic groups; creating enrolment-stage channels for student voice, such as surveys capturing activity preferences and participation barriers (Brereton & Mistry, 2019); diversifying student leadership so that club and society leaders reflect the broader student body’s cultural composition; and simplifying access procedures, since unclear or burdensome administrative processes are known to discourage engagement (Moynihan et al., 2015; Woelert, 2023).

#### 4.4.2. Strategy 2: Mastery-Oriented Sport Environments

The broader literature on motivational climate in sport suggests coaching environment as a plausible lever for psychological outcomes, independent of whatever the present study’s own measurement limitations allow it to establish about sport and stress appraisal. Coach-training interventions promoting mastery-oriented climates have been shown to reduce athlete anxiety relative to untrained-coach controls (R. E. Smith et al., 2007), and empowering coaching climates combining task-involvement, autonomy support, and social support are associated with better athlete health, lower burnout, and fewer physical health symptoms (Appleton & Duda, 2016; Duda, 2013), with coach autonomy support predicting athlete wellbeing across sport settings more broadly (Mossman et al., 2022). We recommend training university sport coordinators and coaches in mastery-oriented practice within existing competitive structures, alongside offering inclusive, non-competitive exercise programmes as an alternative entry point for students discouraged from organised sport by controlling or ego-involving climates (Castillo-Jiménez et al., 2022), fear of failure (Moreno-Murcia et al., 2019), or unfamiliarity with available activities (Young et al., 2003). As these interventions were not implemented or evaluated in the present study, their effectiveness requires direct testing in future longitudinal or experimental research.

#### 4.4.3. Strategy 3: Peer Mentoring Schemes Targeting Loneliness

Loneliness showed among the strongest associations with poor mental health in the present study, yet none of the three engagement forms showed a reliable, adjusted association with it, suggesting general activity provision does not, by itself, reach students experiencing loneliness. Structured peer-to-peer mentoring, academic group work generating repeated informal contact, and residential arrangements facilitating cross-cultural interaction have each shown promise for reducing student loneliness specifically (Shook & Clay, 2012), distinct from the identity-based mechanisms underlying extracurricular belonging. We recommend universities invest in dedicated peer mentoring infrastructure, particularly targeting newly arrived international students facing acculturative stress (R. A. Smith & Khawaja, 2011; Sümer et al., 2011), rather than assuming existing club or activity provision will reach students who are already socially withdrawn. This strategy was not tested in the present study and requires direct evaluation.

#### 4.4.4. Strategy 4: Gender-Differentiated Exercise Provision

General exercise showed a significant direct association with flourishing that was robust to covariate adjustment and was among the more accessible engagement forms overall. It was also associated with lower depression and anxiety in the unadjusted primary model, though these associations were not robust to covariate adjustment or to a higher activity threshold; the flourishing finding is therefore the more stable basis for the strategy below. These findings should be read alongside the broader meta-analytic literature linking physical activity to depression, anxiety, and wellbeing among university students (Liu et al., 2025; Donnelly et al., 2024).

However, women’s exercise participation rate (58.8%) was notably lower than men’s (74.2%) in the present sample, despite women reporting significantly higher depression and anxiety independent of engagement type (Supplementary Table S8), a pattern that sharpens rather than resolves the equity concern raised throughout this paper. This is consistent with evidence that psychological barriers such as self-consciousness and perceived incompetence are common obstacles to physical activity among university students generally (Silva et al., 2022), compounded for women by cultural barriers where mixed-gender or competitive activity carries social stigma (Caperchione et al., 2009). A plausible explanation warranting direct empirical assessment is that gender-differentiated physical activity spaces, privacy-conscious scheduling, and culturally familiar activity formats may be necessary preconditions for exercise’s benefits to be available to all students in this context.

Across all four strategies, Self-Determination Theory (Deci & Ryan, 2000) offers a unifying frame: engagement is most likely when environments provide relatedness without requiring prior social ties, competence without inviting social comparison, and autonomy without pressure. Participation rates did not differ meaningfully by nationality in the present sample (extracurricular, sport, and exercise all within five percentage points of each other by citizenship status; Supplementary Table S2), suggesting citizenship status alone is a coarse predictor of engagement; this does not indicate international students face no barriers, but that the cultural representation emphasised in Strategy 1 may matter more than nationality per se. Universities that treat diverse student wellbeing as a design problem, asking which structural choices make activities genuinely accessible across backgrounds, are more likely to distribute the benefits of campus engagement equitably than those that simply increase provision.

### 4.5. Limitations

The cross-sectional design precludes causal inference; students who feel greater social belonging may be more likely to participate in extracurricular activities, reversing the direction of the relationship hypothesised here, and longitudinal research is needed to establish directionality. Participation was operationalised as a binary variable, which does not capture meaningful variation in frequency, quality, or type of engagement within each category. The Stress Mindset Measure showed poor psychometric coherence in this sample: a confirmatory factor analysis indicated its positive and negative items form statistically independent subscales rather than a single coherent construct (Supplementary Table S3), and stress mindset findings are treated as exploratory throughout. Participants excluded from the path model due to missing predictor data reported significantly lower depression than included participants, suggesting the analytic sample may modestly overrepresent students with elevated depressive symptoms; this did not materially affect the primary findings under a multiple imputation sensitivity analysis (Supplementary Table S10).

The exercise threshold distinguished students reporting less than 1 h of weekly exercise from those reporting at least 1 h; this is not a sedentary classification, as sedentary behaviour was not directly measured, nor does it represent compliance with or departure from established physical activity guidelines such as the WHO’s 150 min/week recommendation, and a higher-threshold sensitivity analysis showed the exercise associations with depression and anxiety weakened further at this higher level (Supplementary Table S5). These associations were also partly confounded by gender: adjusting for gender, age, and institution attenuated exercise’s associations with depression and anxiety to non-significance (Supplementary Table S8), reflecting the substantially higher rate of exercise participation among men and significantly higher depression and anxiety among women independent of engagement type. The sample is drawn from English-medium institutions in only two Emirates; generalisability to Arabic-medium or other Arab Gulf higher education contexts requires direct investigation. The predominantly female sample and binary operationalisation of exercise preclude examination of gender differences in exercise type, setting, or perceived barriers, which may be particularly salient in culturally conservative contexts. Subgroup analyses by degree level and gender were broadly consistent with the primary findings but were likely underpowered, particularly for sport comparisons within the small postgraduate subgroup, and should not be read as a substitute for adequately powered stratified analysis. Prior sport or exercise experience prior to university enrolment was not assessed and may influence both current engagement patterns and the psychological correlates examined here. Formal psychometric validation of the study measures in UAE or culturally diverse samples has not been established (see Section 2.2 Measures).

### 4.6. Conclusions

Campus engagement is associated with student wellbeing, but not uniformly and not through equivalent correlates. Extracurricular participation is associated with social belonging, which in turn is associated with higher flourishing and lower depression and anxiety; this is the strongest and most consistent association identified in the study. Loneliness remains strongly associated with poorer mental health, and none of the three engagement forms showed a reliable adjusted association with it, pointing to a gap in how universities currently approach social wellbeing support. Sport showed a small association with adaptive stress appraisal and anxiety; given the stress mindset measure’s poor reliability in this sample, this remains an unconfirmed pattern rather than an established finding and requires replication with a more reliable measure before it can inform practice. Exercise shows the most direct association with flourishing, with additional associations with depression and anxiety that occurred only in the unadjusted primary model and were not robust to covariate adjustment or threshold sensitivity, and is among the more accessible forms of campus wellbeing support. Because the design is cross-sectional, none of these associations should be read as demonstrating that engagement causes changes in wellbeing; longitudinal and intervention research is needed to test directionality. These associations were identified in a sample that was predominantly undergraduate and predominantly female, and educational level and gender were not stratified in the primary analyses; the reported associations should therefore be read as characterising the sample as a whole rather than any specific subgroup within it.

The challenge for universities in diverse, internationally enrolled contexts is not simply to offer these activities but to understand how they might be designed to reach the students who need them most. This requires understanding which activities are effective, through which mechanisms, for which students, and under what conditions. The present study offers an important foundation for advancing that understanding.

## Figures and Tables

**Table 1:** Descriptive statistics and bivariate correlations among study variables.

Variable	*M*	*SD*	1	2	3	4	5	6
1. Social Belonging	2.88	1.09	—					
2. Stress Mindset	10.09	2.65	0.13 ***	—				
3. Loneliness	6.16	1.91	−0.48 ***	−0.10 **	—			
4. Flourishing	41.08	8.61	0.48 ***	0.14 ***	−0.39 ***	—		
5. Depression (PHQ-9)	11.94	7.14	−0.35 ***	−0.13 ***	0.44 ***	−0.44 ***	—	
6. Anxiety (GAD-7)	10.24	6.04	−0.28 ***	−0.17 ***	0.38 ***	−0.33 ***	0.70 ***	—

Note. *N* ranges from 673 to 801 due to missing data across variables. ** *p* < 0.01. *** *p* < 0.001.

**Table 2:** Group differences in psychological outcomes by form of campus engagement (Welch’s *t*-Test).

Variable	*n*	*M (SD)*	*n*	*M (SD)*	*t(df)*	*p*	*d*
Extracurricular Participation							
	No Participation		Participation				
Social Belonging	287	2.50 (1.07)	384	3.18 (1.01)	−8.25 (597.07)	<0.001	−0.65
Stress Mindset	290	9.84 (2.71)	389	10.27 (2.60)	−2.09 (601.50)	0.037	−0.16
Loneliness	317	6.17 (1.99)	416	6.15 (1.85)	0.15 (653.65)	0.882	0.01
Flourishing	339	39.70 (9.42)	430	42.21 (7.76)	−3.95 (649.77)	<0.001	−0.29
Depression (PHQ-9)	356	12.55 (7.76)	439	11.51 (6.54)	2.02 (694.95)	0.044	0.15
Anxiety (GAD-7)	323	10.42 (6.21)	414	10.11 (5.93)	0.69 (676.49)	0.492	0.05
Sport Participation							
	No Participation		Participation				
Social Belonging	594	2.85 (1.08)	79	3.14 (1.14)	−2.12 (97.45)	0.036	−0.26
Stress Mindset	597	10.00 (2.67)	81	10.81 (2.38)	−2.86 (109.21)	0.005	−0.32
Loneliness	647	6.18 (1.93)	89	6.03 (1.79)	0.70 (117.99)	0.486	0.08
Flourishing	685	40.83 (8.72)	88	43.07 (7.43)	−2.61 (120.05)	0.010	−0.28
Depression (PHQ-9)	710	11.89 (7.21)	91	12.32 (6.59)	−0.58 (119.41)	0.563	−0.06
Anxiety (GAD-7)	655	10.34 (6.06)	84	9.44 (5.93)	1.31 (106.44)	0.193	0.15
Exercise Participation							
	Low Exercise (<1 h/wk)		Active (≥1 h/wk)				
Social Belonging	237	2.79 (1.11)	426	2.95 (1.08)	−1.81 (476.31)	0.070	−0.15
Stress Mindset	239	10.05 (2.58)	426	10.08 (2.69)	−0.15 (510.11)	0.880	−0.01
Loneliness	260	6.38 (1.86)	472	6.03 (1.93)	2.41 (552.62)	0.016	0.19
Flourishing	261	39.64 (9.19)	467	42.00 (8.17)	−3.46 (487.29)	<0.001	−0.27
Depression (PHQ-9)	264	13.42 (6.94)	472	11.92 (6.74)	2.83 (531.31)	0.005	0.22
Anxiety (GAD-7)	262	11.05 (6.15)	462	9.73 (5.93)	2.82 (525.58)	0.005	0.22

Note. Welch’s *t*-test reported throughout to account for unequal group sizes; degrees of freedom appear in parentheses alongside *t*-values. Cohen’s *d* uses unpooled standard deviations and is computed as (*M* non-participating − *M* participating)/*SD*; negative values indicate higher scores among participants. Three comparisons (extracurricular–stress mindset, extracurricular–depression, sport–belonging) did not survive FDR correction across all 18 comparisons and should be interpreted as descriptive (see Supplementary Table S6 for corrected *p*-values and 95% CIs). Low exercise = less than 1 h per week of moderate-to-vigorous physical activity; Active = 1 or more hours per week.

**Table 3:** Trimmed path model: standardised and unstandardised estimates with bootstrapped 95% confidence intervals (*N* = 733).

						Bootstrap 95% CI
Path	*B*	*SE*	β	z	*p*	*[LL, UL]*
Model fit: χ^2^(12) = 20.08, *p* = 0.066; CFI = 0.994; TLI = 0.983; RMSEA = 0.030 [0.000, 0.053]; SRMR = 0.020						
A-paths: Predictors → Mediators						
Extracurricular → Social Belonging	0.658	0.071	0.300	9.33	<0.001	[0.519, 0.797]
Exercise → Loneliness	−0.220	0.130	−0.055	−1.70	0.090	[−0.476, 0.037]
Sport → Stress Mindset	0.835	0.310	0.103	2.69	0.007	[0.227, 1.443]
B-paths: Mediators → Outcomes						
Social Belonging → Flourishing	2.952	0.298	0.373	9.91	<0.001	[2.367, 3.537]
Loneliness → Flourishing	−0.930	0.165	−0.206	−5.64	<0.001	[−1.254, −0.606]
Social Belonging → Depression	−1.209	0.241	−0.192	−5.01	<0.001	[−1.681, −0.737]
Loneliness → Depression	1.215	0.134	0.339	9.10	<0.001	[0.953, 1.477]
Stress Mindset → Depression	−0.150	0.085	−0.058	−1.76	0.078	[−0.317, 0.017]
Social Belonging → Anxiety	−0.644	0.222	−0.116	−2.90	0.004	[−1.079, −0.209]
Loneliness → Anxiety	0.950	0.123	0.301	7.71	<0.001	[0.709, 1.191]
Stress Mindset → Anxiety	−0.251	0.081	−0.110	−3.10	0.002	[−0.410, −0.092]
Direct paths (C′)						
Exercise → Flourishing	1.594	0.576	0.089	2.77	0.006	[0.465, 2.723]
Sport → Depression	1.086	0.518	0.052	2.10	0.036	[0.071, 2.101]
Exercise → Depression	−1.023	0.470	−0.072	−2.18	0.029	[−1.944, −0.102]
Exercise → Anxiety	−0.904	0.427	−0.072	−2.12	0.034	[−1.741, −0.067]
Bootstrapped indirect effects						
Extracurricular → Social Belonging → Flourishing	1.942	0.296	0.112	6.56	<0.001	[1.422, 2.584]
Extracurricular → Social Belonging → Depression	−0.796	0.175	−0.058	−4.54	<0.001	[−1.178, −0.480]
Extracurricular → Social Belonging → Anxiety	−0.424	0.147	−0.035	−2.89	0.004	[−0.717, −0.141]
Exercise → Loneliness → Flourishing	0.205	0.129	0.011	1.58	0.114	[−0.023, 0.503]
Exercise → Loneliness → Depression	−0.267	0.159	−0.019	−1.68	0.093	[−0.603, 0.046]
Exercise → Loneliness → Anxiety	−0.209	0.127	−0.017	−1.65	0.100	[−0.483, 0.034]
Sport → Stress Mindset → Depression	−0.125	0.088	−0.006	−1.43	0.154	[−0.353, 0.009]
Sport → Stress Mindset → Anxiety	−0.210	0.096	−0.011	−2.19	0.028	[−0.446, −0.063]

Note. *N* = 733. Model estimated using maximum likelihood with full information maximum likelihood (FIML) for missing data. Bootstrap confidence intervals based on 5000 resamples (bias-corrected accelerated). β = standardised estimate. B = unstandardised estimate. LL = lower limit; UL = upper limit. Predictors: Sport (0 = no participation, 1 = participation); Extracurricular (0 = no participation, 1 = participation); Exercise (0 = less than 1 h/week, 1 = 1 or more hours/week). Indirect effects through Exercise → Loneliness are non-significant under bootstrapping (CIs include zero). Paths reported with *p* < 0.10 included in model for theoretical completeness.

## Data Availability

The R analysis code and summary-level data supporting the findings of this study are openly available on the Open Science Framework at https://doi.org/10.17605/OSF.IO/DFWQR. Anonymised individual-level data are available from the corresponding author (cbryan@aus.edu) upon reasonable request.

## Supplementary Materials

The following supporting information can be downloaded at: https://www.mdpi.com/article/10.3390/ejihpe16080116/s1. Table S1: Group Differences by Degree Level (Undergraduate vs. Postgraduate); Table S2: Group Differences by Gender (Men vs. Women); Table S3: Confirmatory Factor Analysis and Reliability for the Stress Mindset Measure and Social Belonging Composite; Table S4: Overlap Among the Three Campus Engagement Variables; Table S5: Exercise Threshold Sensitivity Analysis; Table S6: False Discovery Rate (FDR)-Corrected p Values and 95% Confidence Intervals for Cohen’s d Across All 18 Group Comparisons; Table S7: Full Saturated Path Model, R^2^, and Predictor Covariances; Table S8: Covariate-Adjusted Path Model (Gender, Age, and Institution); Table S9: Comparison of Maximum Likelihood and Robust Maximum Likelihood (MLR) Estimation; Table S10: Multiple Imputation Sensitivity Analysis; Table S11: Variable-Level Missingness; Table S12: Comparison of Participants Included in and Excluded from the Primary Path Model; Table S13: Distributional Properties and Screening Cut-off Proportions for PHQ-9 and GAD-7; Supplementary File S1: Complete lavaan Model Syntax (Saturated and Trimmed Models).
