# Supplementary material for: Pathways to Wellbeing: Differentiated Associations Across Three Forms of Campus Engagement in a Diverse UAE University Context
Source: Eur J Investig Health Psychol Educ. 2026 Aug 12;16(8):116. doi: 10.3390/ejihpe16080116 (PMC13512712; doi:10.3390/ejihpe16080116)
Supplement: Supplementary file 1 [file ejihpe-16-00116-s001.zip › ejihpe-4411388-supplementary.pdf]

Supplementary Materials

Three Pathways to Wellbeing: Extracurricular Participation, Sport, and Exercise as Distinct Contributors to Flourishing and Mental Health in a Diverse UAE University Context

This document contains 13 supplementary tables (S1–S13) and Appendix B (complete lavaan model syntax). Analyses were conducted in R (version 4.2.1) using the packages lavaan (0.6-12), psych (2.3.9), effectsize (1.0.1), semTools (0.5-6), mice (3.16.0), and polycor. All models used full information maximum likelihood (FIML) to handle missing data unless otherwise noted.

Contents List

Table	Title
**S1**	Group Differences by Degree Level (Undergraduate vs. Postgraduate)
**S2**	Group Differences by Gender (Men vs. Women)
**S3**	Confirmatory Factor Analysis and Reliability for the Stress Mindset Measure and Social Belonging Composite
**S4**	Overlap Among the Three Campus Engagement Variables
**S5**	Exercise Threshold Sensitivity Analysis
**S6**	False Discovery Rate (FDR)-Corrected *p* Values and 95% Confidence Intervals for Cohen's *d* Across All 18 Group Comparisons
**S7**	Full Saturated Path Model, R^2, and Predictor Covariances
**S8**	Covariate-Adjusted Path Model (Gender, Age, and Institution)
**S9**	Comparison of Maximum Likelihood and Robust Maximum Likelihood (MLR) Estimation
**S10**	Multiple Imputation Sensitivity Analysis
**S11**	Variable-Level Missingness
**S12**	Comparison of Participants Included in and Excluded from the Primary Path Model
**S13**	Distributional Properties and Screening Cut-off Proportions for PHQ-9 and GAD-7
**File S1**	Complete lavaan Model Syntax (Saturated and Trimmed Models)

Table S1

Group Differences by Degree Level (Undergraduate vs. Postgraduate)

Comparison	*Outcome*	*n (No)*	*M (No)*	*SD (No)*	*n (Yes)*	*M (Yes)*	*SD (Yes)*	*t*	*df*	*p*	*d*
**Undergraduate (n = 723)**											
Extracurricular	Belonging	255	2.47	1.08	354	3.15	1.03	7.74	530.9	<.001	0.64
Extracurricular	Stress Mindset	255	9.86	2.64	358	10.29	2.52	2.00	531.5	.046	0.16
Extracurricular	Loneliness	284	6.23	1.98	383	6.17	1.84	-0.35	584.2	.725	-0.03
Extracurricular	Flourishing	300	39.38	9.43	395	41.95	7.88	3.82	576.8	<.001	0.30
Extracurricular	Depression	314	12.9	7.6	403	11.82	6.43	-2.01	611.3	.045	-0.15
Extracurricular	Anxiety	291	10.83	6.18	382	10.33	5.85	-1.06	606.0	.289	-0.08
Sport	Belonging	535	2.83	1.09	76	3.11	1.14	2.05	95.3	.043	0.26
Sport	Stress Mindset	537	10.01	2.59	78	10.85	2.39	2.86	105.2	.005	0.34
Sport	Loneliness	585	6.22	1.92	85	6.05	1.79	-0.81	114.1	.418	-0.09
Sport	Flourishing	615	40.53	8.77	84	42.90	7.50	2.66	116.4	.009	0.29
Sport	Depression	637	12.21	7.06	86	12.60	6.54	0.52	113.5	.601	0.06
Sport	Anxiety	595	10.67	6.00	80	9.54	5.88	-1.61	102.4	.110	-0.19
Exercise	Belonging	217	2.76	1.12	387	2.92	1.09	1.69	438.4	.092	0.14
Exercise	Stress Mindset	218	10.12	2.54	387	10.1	2.61	-0.12	461.0	.906	-0.01
Exercise	Loneliness	237	6.42	1.85	429	6.07	1.93	-2.28	504.3	.023	-0.18
Exercise	Flourishing	238	39.42	9.22	424	41.72	8.25	3.19	447.3	.002	0.26
Exercise	Depression	241	13.46	6.85	429	12.30	6.62	-2.14	483.2	.033	-0.17
Exercise	Anxiety	240	11.32	6.12	420	10.04	5.87	-2.61	480.7	.009	-0.21

Comparison	*Outcome*	*n (No)*	*M (No)*	*SD (No)*	*n (Yes)*	*M (Yes)*	*SD (Yes)*	*t*	*df*	*p*	*d*
**Postgraduate (n = 75)**											
Extracurricular	Belonging	31	2.75	1.00	28	3.53	0.79	3.32	56.2	.002	0.86
Extracurricular	Stress Mindset	31	9.65	3.29	29	10.21	3.35	0.65	57.6	.516	0.17
Extracurricular	Loneliness	33	5.70	2.01	31	5.68	1.81	-0.04	61.9	.967	-0.01
Extracurricular	Flourishing	38	42.29	9.21	33	45.73	4.90	2.00	57.9	.050	0.47
Extracurricular	Depression	41	10.15	8.47	34	7.32	6.49	-1.63	72.6	.107	-0.37
Extracurricular	Anxiety	32	6.75	5.21	30	6.87	5.98	0.08	57.6	.935	0.02
Sport	Depression	70	8.97	7.85	5	7.40	5.98	-0.55	5.0	.603	-0.23
Exercise	Belonging	19	2.99	1.06	38	3.20	0.97	0.73	33.3	.473	0.21
Exercise	Stress Mindset	20	9.5	2.84	38	9.87	3.40	0.44	45.3	.663	0.12
Exercise	Loneliness	22	5.91	1.87	42	5.57	1.93	-0.68	43.8	.501	-0.18
Exercise	Flourishing	22	42.45	8.42	42	44.86	6.84	1.15	35.9	.256	0.31
Exercise	Depression	22	12.55	7.96	42	7.98	6.86	-2.29	37.6	.028	-0.62
Exercise	Anxiety	21	7.76	5.76	41	6.32	5.44	-0.95	38.4	.347	-0.26

Note. All comparisons used Welch's independent-samples t-test. Sport comparisons within the postgraduate subgroup other than Depression are omitted due to insufficient cell sizes (fewer than 5 sport-participating postgraduate students on most outcomes). Participation rates by degree level: undergraduate — sport 11.9%, extracurricular 56.2%, exercise 64.0%; postgraduate — sport 6.7%, extracurricular 45.3%, exercise 65.6%. Results should be interpreted with caution given the small postgraduate subgroup (n = 75).

Table S2

Group Differences by Gender (Men vs. Women)

Comparison	*Outcome*	*n (No)*	*M (No)*	*SD (No)*	*n (Yes)*	*M (Yes)*	*SD (Yes)*	*t*	*df*	*p*	*d*
**Men (n = 271)**											
Extracurricular	Belonging	111	2.41	1.06	123	3.03	1.04	4.53	228.8	<.001	0.59
Extracurricular	Stress Mindset	112	9.51	3	127	10.59	2.62	2.95	222.3	.003	0.38
Extracurricular	Loneliness	120	6.11	1.9	132	6.14	1.89	0.12	247.6	.907	0.01
Extracurricular	Flourishing	127	39.41	9.83	134	43.83	7.75	4.02	239.5	<.001	0.50
Extracurricular	Depression	132	11.59	7.61	138	10.39	6.68	-1.37	260.1	.171	-0.17
Extracurricular	Anxiety	122	8.87	5.81	130	8.42	6.12	-0.59	250	.553	-0.07
Sport	Belonging	195	2.69	1.06	39	2.97	1.23	1.34	50	.187	0.25
Sport	Stress Mindset	198	9.78	2.87	41	11.54	2.28	4.27	68.9	<.001	0.68
Sport	Loneliness	207	6.14	1.92	45	6.07	1.79	-0.23	67.8	.819	-0.04
Sport	Flourishing	217	41.31	9.22	45	43.47	8.1	1.58	69.8	.118	0.25
Sport	Depression	224	10.94	7.29	47	10.94	6.72	-0.01	70.6	.996	0.00
Sport	Anxiety	209	8.72	5.85	43	8.26	6.53	-0.43	56.7	.669	-0.07
Exercise	Belonging	62	2.57	1.12	168	2.8	1.08	1.39	105.5	.166	0.21
Exercise	Stress Mindset	61	9.82	2.63	172	10.12	2.89	0.74	115	.463	0.11
Exercise	Loneliness	64	6.31	2.04	187	6.05	1.85	-0.9	100.6	.371	-0.13
Exercise	Flourishing	64	40.58	10.46	185	41.94	8.56	0.94	93.9	.350	0.14
Exercise	Depression	65	12.62	7.81	187	10.93	6.65	-1.55	98.1	.123	-0.23
Exercise	Anxiety	65	8.91	6.53	183	8.52	5.79	-0.42	102	.677	-0.06

Comparison	*Outcome*	*n (No)*	*M (No)*	*SD (No)*	*n (Yes)*	*M (Yes)*	*SD (Yes)*	*t*	*df*	*p*	*d*
**Women (n = 529)**											
Extracurricular	Belonging	176	2.56	1.08	260	3.24	1	6.67	355.3	<.001	0.66
Extracurricular	Stress Mindset	175	10.05	2.5	261	10.11	2.58	0.24	381.4	.809	0.02
Extracurricular	Loneliness	197	6.21	2.04	283	6.16	1.83	-0.29	391.9	.772	-0.03
Extracurricular	Flourishing	212	39.88	9.19	295	41.47	7.68	2.06	403	.040	0.19
Extracurricular	Depression	224	13.11	7.81	300	12.02	6.43	-1.71	424.9	.088	-0.15
Extracurricular	Anxiety	201	11.37	6.26	283	10.89	5.7	-0.85	405	.395	-0.08
Sport	Belonging	398	2.93	1.08	40	3.31	1.04	2.18	47.9	.034	0.36
Sport	Stress Mindset	398	10.1	2.57	40	10.07	2.28	-0.06	49.5	.952	-0.01
Sport	Loneliness	439	6.2	1.93	44	6	1.8	-0.68	53.4	.498	-0.10
Sport	Flourishing	467	40.6	8.49	43	42.65	6.73	1.87	55.1	.067	0.27
Sport	Depression	485	12.33	7.15	44	13.8	6.18	1.49	54	.142	0.22
Sport	Anxiety	445	11.11	6.01	41	10.68	5.02	-0.51	51.2	.611	-0.08
Exercise	Belonging	175	2.86	1.1	257	3.04	1.07	1.67	367	.096	0.16
Exercise	Stress Mindset	178	10.13	2.56	253	10.05	2.55	-0.31	379.5	.756	-0.03
Exercise	Loneliness	196	6.41	1.8	284	6.02	1.99	-2.22	445.7	.027	-0.20
Exercise	Flourishing	197	39.33	8.75	281	42.04	7.93	3.46	395.1	.001	0.32
Exercise	Depression	199	13.68	6.63	284	12.57	6.75	-1.79	431	.075	-0.16
Exercise	Anxiety	197	11.76	5.87	278	10.53	5.9	-2.26	423.6	.025	-0.21

Note. All comparisons used Welch's independent-samples t-test. The sport–stress mindset association was significant among men ($d = 0.68$, $p < .001$) but not among women ($d = -0.01$, $p = .952$), suggesting this pathway may operate differently across genders. Participation rates: men — sport 17.3%, extracurricular 51.1%, exercise 74.2%; women — sport 8.3%, extracurricular 57.3%, exercise

58.8%. Participation rates by expatriate status: UAE nationals — sport 12.4%, extracurricular 54.2%, exercise 62.1%; non-UAE nationals — sport 10.4%, extracurricular 56.4%, exercise 65.2%.

Table S3

Confirmatory Factor Analysis and Reliability for the Stress Mindset Measure and Social Belonging Composite

Reliability (Cronbach's Alpha and McDonald's Omega) for All Scales

Scale	*Items*	*n*	*α*	*ω*
Flourishing Scale	8	773	0.868	0.871
Depression (PHQ-9)	9	748	0.858	0.860
Anxiety (GAD-7)	7	739	0.912	0.914
Social Belonging	4	673	0.749	0.761
Loneliness (UCLA-3)	3	736	0.843	0.844
Stress Mindset (SMM)	4	678	0.399	0.544

SMM Single-Factor CFA

Item	*Standardised Loading*	*p*
STRESSMINDSET_1R	0.138	—
STRESSMINDSET_2	-0.772	.002
STRESSMINDSET_3R	-0.142	.021
STRESSMINDSET_4	-0.823	.003

Note. Single-factor fit: $\chi^2(2) = 23.86$, $p < .001$; CFI = .944; TLI = .832; RMSEA = .127, 90% CI [.084, .175]; SRMR = .057. The reversed items (1R, 3R) loaded weakly and near zero relative to the non-reversed items, indicating the four items do not form a coherent single factor.

SMM Two-Factor CFA (Positive vs. Negative Subscales)

The two-factor model (positive items: STRESSMINDSET_2, STRESSMINDSET_4; negative/reversed items: STRESSMINDSET_1R, STRESSMINDSET_3R) is just-identified (df = 0); fit is perfect by construction and not informative. The estimated correlation between the two latent factors was $r = -.001$, indicating the positive and negative subscales are statistically independent in this sample. Corrected item-total correlations from the reliability analysis were:

STRESSMINDSET_1R, r = −.014; STRESSMINDSET_2, r = .665; STRESSMINDSET_3R, r = .284; STRESSMINDSET_4, r = .654. The negative item-total correlation for item 1R indicates this item does not cohere with the remaining items in the direction expected for a summed score.

Social Belonging Composite CFA

Item	*Standardised Loading*	*p*
SENSESOCACAFIT_1	0.724	—
SENSESOCACAFIT_2	0.698	<.001
SENSESOCACAFIT_3	0.397	<.001
COHESIONSCALE_1	0.807	<.001

Note. Single-factor fit: $\chi^2(2) = 44.68$, $p < .001$; CFI = .940; TLI = .821; RMSEA = .178, 90% CI [.135, .225]; SRMR = .051. Corrected item-total correlations: SENSESOCACAFIT_1, r = .669; SENSESOCACAFIT_2, r = .721; SENSESOCACAFIT_3, r = .424; COHESIONSCALE_1, r = .746. All four loadings were positive and of acceptable magnitude, supporting the coherence of the belonging composite despite the elevated RMSEA, which is a known artifact of low degrees of freedom (df = 2) rather than evidence of poor model fit (Kenny, Kaniskan, & McCoach, 2015). Given the poor psychometric performance of the Stress Mindset Measure documented above, all findings involving stress mindset are presented as exploratory and tentative throughout the manuscript, retained primarily for comparability with prior research using the same instrument.

Table S4

Overlap Among the Three Campus Engagement Variables

Combination Frequencies

Combination	*n*	*%*
Extracurricular + Exercise (no sport)	198	27.0
Exercise only	195	26.6
Extracurricular only	130	17.7
None of the three	121	16.5
All three	78	10.6
Sport + Extracurricular (no exercise)	11	1.5

Tetrachoric Correlations

Variable	*1*	*2*	*3*
1. Sport	—		
2. Extracurricular	.324	—	
3. Exercise	.181	.046	—

Note. No student reported sport participation without also reporting extracurricular participation (n = 0 in the Sport-only and Sport + Exercise-only cells beyond what is shown), because the extracurricular item explicitly includes "sports" in its wording. The simultaneous path model therefore estimates sport's association over and above general extracurricular involvement. The zero-order Pearson correlation between sport participation and depression was $r = .019$, $t(799) = 0.54$, $p = .589$, indicating the small positive adjusted path from sport to depression in the primary model ($\beta = .052$) reflects a suppression effect rather than a meaningful raw association.

Table S5

Exercise Threshold Sensitivity Analysis

Path	*β (Original, ≥1 hr/wk)*	*p*	*β (Sensitivity, ≥2–3 hrs/wk)*	*p*
Extracurricular → Belonging	0.3	<.001	0.299	<.001
Exercise → Loneliness	-0.055	.090	-0.038	.239
Sport → Stress Mindset	0.103	.007	0.103	.007
Belonging → Flourishing	0.373	<.001	0.374	<.001
Loneliness → Flourishing	-0.206	<.001	-0.209	<.001
Exercise → Flourishing	0.089	.006	0.062	.054
Belonging → Depression	-0.192	<.001	-0.194	<.001
Loneliness → Depression	0.339	<.001	0.342	<.001
Stress Mindset → Depression	-0.058	.078	-0.057	.084
Sport → Depression	0.052	.036	0.049	.049
Exercise → Depression	-0.072	.029	-0.033	.314
Belonging → Anxiety	-0.116	.004	-0.119	.003
Loneliness → Anxiety	0.301	<.001	0.303	<.001
Stress Mindset → Anxiety	-0.11	.002	-0.109	.002
Exercise → Anxiety	-0.072	.034	-0.037	.282

Note. Original threshold: low = <1 hour/week (n = 264), active = ≥1 hour/week (n = 472, 64.1%). Sensitivity threshold: low = <2–3 hours/week (n = 433), active = ≥2–3 hours/week (n = 303, 41.2%), more closely approximating the World Health Organization recommendation of 150 minutes of moderate-intensity activity per week. The extracurricular and sport paths were unaffected by the threshold change. The exercise associations with loneliness and flourishing remained directionally consistent but attenuated (flourishing weakened to marginal significance, p = .054), and the exercise associations with depression and anxiety lost statistical significance at the higher threshold. This indicates that exercise's direct symptom-reduction associations are most robust at lower activity thresholds and should be interpreted as sensitive to the specific threshold selected.

Table S6

False Discovery Rate (FDR)-Corrected p Values and 95% Confidence Intervals for Cohen's d Across All 18 Group Comparisons

Comparison	*Outcome*	*d*	*95% CI*	*p (Raw)*	*p (FDR)*
Extracurricular	Belonging	0.647	[0.489, 0.804]	<.001	<.001
Extracurricular	Stress Mindset	0.163	[0.010, 0.316]	.037	.067
Extracurricular	Loneliness	-0.011	[-0.158, 0.136]	.882	.882
Extracurricular	Flourishing	0.29	[0.145, 0.435]	<.001	.001
Extracurricular	Depression	-0.145	[-0.286, -0.004]	.044	.072
Extracurricular	Anxiety	-0.051	[-0.197, 0.095]	.492	.590
Sport	Belonging	0.26	[0.017, 0.501]	.036	.067
Sport	Stress Mindset	0.323	[0.097, 0.548]	.005	.015
Sport	Loneliness	-0.077	[-0.292, 0.139]	.486	.590
Sport	Flourishing	0.276	[0.065, 0.487]	.010	.027
Sport	Depression	0.062	[-0.148, 0.273]	.563	.634
Sport	Anxiety	-0.151	[-0.377, 0.076]	.193	.267
Exercise	Belonging	0.148	[-0.012, 0.307]	.070	.106
Exercise	Stress Mindset	0.012	[-0.145, 0.170]	.880	.882
Exercise	Loneliness	-0.185	[-0.336, -0.034]	.016	.036
Exercise	Flourishing	0.272	[0.117, 0.427]	<.001	.004
Exercise	Depression	-0.218	[-0.370, -0.067]	.005	.015
Exercise	Anxiety	-0.219	[-0.372, -0.066]	.005	.015

Note. Cohen's d calculated using unweighted (unpooled) standard deviations for consistency with Welch's t-test, which does not assume equal variances. *p* (FDR) reflects Benjamini-Hochberg false discovery rate correction (Benjamini & Hochberg, 1995) applied across all 18 comparisons. Three comparisons that were significant at the conventional $\alpha = .05$ threshold before correction — extracurricular–stress mindset, extracurricular–depression, and sport–belonging — did not survive FDR correction and should be interpreted as descriptive rather than confirmed group differences. All other significant comparisons in Table 2 of the main manuscript remained significant after correction.

Table S7

Full Saturated Path Model, R^2, and Predictor Covariances

Saturated Model: All Structural Paths

Path	*B*	*SE*	*β*	*z*	*p*
Sport → Belonging	-0.064	0.130	-0.019	-0.49	.622
Extracurricular → Belonging	0.673	0.085	0.306	7.96	<.001
Exercise → Belonging	0.144	0.084	0.063	1.72	.086
Sport → Loneliness	-0.061	0.231	-0.010	-0.26	.793
Extracurricular → Loneliness	0.014	0.15	0.004	0.10	.924
Exercise → Loneliness	-0.325	0.15	-0.082	-2.17	.030
Sport → Stress Mindset	0.699	0.333	0.086	2.10	.036
Extracurricular → Stress Mindset	0.333	0.218	0.062	1.53	.126
Exercise → Stress Mindset	-0.067	0.216	-0.012	-0.31	.754
Belonging → Flourishing	2.785	0.32	0.352	8.71	<.001
Loneliness → Flourishing	-0.945	0.167	-0.209	-5.64	<.001
Stress Mindset → Flourishing	0.204	0.111	0.063	1.83	.067
Sport → Flourishing	0.196	0.909	0.007	0.22	.829
Extracurricular → Flourishing	0.534	0.623	0.031	0.86	.392
Exercise → Flourishing	1.534	0.583	0.085	2.63	.009
Belonging → Depression	-1.028	0.257	-0.163	-4.00	<.001
Loneliness → Depression	1.259	0.136	0.35	9.28	<.001
Stress Mindset → Depression	-0.193	0.089	-0.075	-2.17	.030
Sport → Depression	1.511	0.731	0.072	2.07	.039
Extracurricular → Depression	-0.955	0.504	-0.069	-1.90	.058
Exercise → Depression	-1.035	0.472	-0.072	-2.19	.028
Belonging → Anxiety	-0.707	0.238	-0.127	-2.97	.003
Loneliness → Anxiety	0.929	0.125	0.294	7.41	<.001
Stress Mindset → Anxiety	-0.278	0.082	-0.122	-3.39	<.001
Sport → Anxiety	-0.025	0.679	-0.001	-0.04	.971
Extracurricular → Anxiety	0.342	0.465	0.028	0.74	.462
Exercise → Anxiety	-0.910	0.434	-0.072	-2.10	.036

R^2 by Model

Endogenous Variable	*R^2 (Saturated)*	*R^2 (Trimmed)*
Belonging	0.096	0.090
Loneliness	0.007	0.003
Stress Mindset	0.015	0.011
Flourishing	0.274	0.265
Depression	0.238	0.230
Anxiety	0.172	0.167

Predictor Covariances (Freely Estimated)

Pair	*Covariance*	*r (Standardised)*
Sport – Extracurricular	0.052	0.324
Sport – Exercise	0.028	0.181
Extracurricular – Exercise	0.011	0.046

Note. N = 733 (saturated), 733 (trimmed). Saturated model fit: $\chi^2(0) = 0.000$, df = 0 (just-identified; fit indices not informative). Trimmed model fit: $\chi^2(12) = 20.08$, $p = .066$; CFI = .994; TLI = .983; RMSEA = .030, 90% CI [.000, .053]; SRMR = .020. The complete lavaan syntax for both models is provided in Appendix B.

Table S8
Covariate-Adjusted Path Model (Gender, Age, and Institution)

Path	*B*	*SE*	*β*	*z*	*p*
Extracurricular → Belonging	0.656	0.072	0.299	9.17	<.001
Exercise → Loneliness	-0.199	0.131	-0.05	-1.52	.128
Sport → Stress Mindset	0.831	0.312	0.103	2.67	.008
Belonging → Flourishing	3.022	0.3	0.382	10.08	<.001
Loneliness → Flourishing	-0.899	0.165	-0.199	-5.45	<.001
Exercise → Flourishing	1.428	0.581	0.08	2.46	.014
Belonging → Depression	-1.3	0.24	-0.207	-5.43	<.001
Loneliness → Depression	1.157	0.132	0.322	8.76	<.001
Stress Mindset → Depression	-0.176	0.085	-0.068	-2.08	.038
Sport → Depression	1.272	0.515	0.061	2.47	.014
Exercise → Depression	-0.789	0.468	-0.055	-1.69	.091
Belonging → Anxiety	-0.797	0.216	-0.144	-3.69	<.001
Loneliness → Anxiety	0.902	0.119	0.285	7.56	<.001
Stress Mindset → Anxiety	-0.248	0.079	-0.109	-3.14	.002
Exercise → Anxiety	-0.471	0.418	-0.037	-1.13	.260

Covariate Effects

Outcome	*Covariate*	*B*	*SE*	*β*	*z*	*p*
Belonging	Female	0.18	0.083	0.078	2.16	.031
Belonging	Age	0.021	0.012	0.068	1.81	.071
Belonging	Institution B	-0.014	0.106	-0.005	-0.14	.892
Loneliness	Female	0.041	0.149	0.01	0.28	.783
Loneliness	Age	-0.044	0.021	-0.081	-2.14	.033
Loneliness	Institution B	0.305	0.188	0.061	1.62	.104
Stress Mindset	Female	0.114	0.214	0.02	0.53	.596
Stress Mindset	Age	-0.066	0.03	-0.087	-2.19	.029
Stress Mindset	Institution B	0.457	0.267	0.066	1.71	.087
Flourishing	Female	-1.161	0.59	-0.064	-1.97	.049

Outcome	*Covariate*	*B*	*SE*	*β*	*z*	*p*
Flourishing	Age	0.123	0.081	0.05	1.51	.130
Flourishing	Institution B	0.278	0.733	0.012	0.38	.704
Depression	Female	1.919	0.472	0.133	4.07	<.001
Depression	Age	-0.161	0.065	-0.082	-2.47	.014
Depression	Institution B	1.232	0.588	0.069	2.09	.036
Anxiety	Female	2.557	0.424	0.201	6.03	<.001
Anxiety	Age	-0.155	0.06	-0.09	-2.61	.009
Anxiety	Institution B	-1.492	0.53	-0.095	-2.81	.005

Note. N = 732. Model fit: $\chi^2(12) = 18.10$, p = .113; CFI = .996; TLI = .981; RMSEA = .026, 90% CI [.000, .050]; SRMR = .016. Female coded 0 = man, 1 = woman. Institution coded 0 = Institution A, 1 = Institution B. All fifteen primary structural paths retained the same sign as the unadjusted trimmed model; the exercise associations with depression and anxiety attenuated to non-significance after adjustment for gender, age, and institution, reflecting in part the substantially higher rate of exercise participation among men (74.2%) relative to women (58.8%) and the significantly higher depression and anxiety scores reported by women.

Table S9

Comparison of Maximum Likelihood and Robust Maximum Likelihood (MLR) Estimation

Path	*β (ML)*	*p (ML)*	*β (MLR)*	*p (MLR)*
Extracurricular → Belonging	0.300	<.001	0.300	<.001
Exercise → Loneliness	-0.055	.090	-0.055	.089
Sport → Stress Mindset	0.103	.007	0.103	.003
Belonging → Flourishing	0.373	<.001	0.373	<.001
Loneliness → Flourishing	-0.206	<.001	-0.206	<.001
Exercise → Flourishing	0.089	.006	0.089	.008
Belonging → Depression	-0.192	<.001	-0.192	<.001
Loneliness → Depression	0.339	<.001	0.339	<.001
Stress Mindset → Depression	-0.058	.078	-0.058	.091
Sport → Depression	0.052	.036	0.052	.023
Exercise → Depression	-0.072	.029	-0.072	.031
Belonging → Anxiety	-0.116	.004	-0.116	.004
Loneliness → Anxiety	0.301	<.001	0.301	<.001
Stress Mindset → Anxiety	-0.110	.002	-0.110	.002
Exercise → Anxiety	-0.072	.034	-0.072	.037

Note. N = 733, MLR fit: robust $\chi^2(12) = 20.23$, p = .063 (scaling correction factor = 0.992); robust CFI = .993; robust TLI = .982; robust RMSEA = .031, 90% CI [.000, .053]; SRMR = .020. Every structural path retained an identical standardised coefficient and consistent significance pattern under robust estimation, indicating the primary findings are not sensitive to violations of multivariate normality in the PHQ-9 and GAD-7 distributions.

Table S10

Multiple Imputation Sensitivity Analysis

Path	*B (FIML)*	*p (FIML)*	*B (MI, Pooled)*	*p (MI, Pooled)*
Extracurricular → Belonging	0.658	<.001	0.638	<.001
Exercise → Loneliness	-0.220	.089	-0.212	.108
Sport → Stress Mindset	0.835	.003	0.829	.008
Belonging → Flourishing	2.952	<.001	2.898	<.001
Loneliness → Flourishing	-0.930	<.001	-0.906	<.001
Exercise → Flourishing	1.594	.008	1.573	.008
Belonging → Depression	-1.209	<.001	-1.087	<.001
Loneliness → Depression	1.215	<.001	1.324	<.001
Stress Mindset → Depression	-0.15	.091	-0.160	.069
Sport → Depression	1.086	.023	1.216	.029
Exercise → Depression	-1.023	.031	-0.988	.049
Belonging → Anxiety	-0.644	.004	-0.550	.014
Loneliness → Anxiety	0.950	<.001	1.025	<.001
Stress Mindset → Anxiety	-0.251	.002	-0.269	.001
Exercise → Anxiety	-0.904	.037	-0.884	.047

Pooled Indirect Effects

Indirect Effect	*B (Pooled)*	*SE*	*p*
Extracurricular → Belonging → Flourishing	1.849	0.284	<.001
Extracurricular → Belonging → Depression	-0.693	0.178	<.001
Extracurricular → Belonging → Anxiety	-0.351	0.148	.018
Exercise → Loneliness → Flourishing	0.192	0.122	.117
Exercise → Loneliness → Depression	-0.281	0.184	.127
Exercise → Loneliness → Anxiety	-0.218	0.139	.118
Sport → Stress Mindset → Depression	-0.130	0.085	.129
Sport → Stress Mindset → Anxiety	-0.221	0.104	.034

Note. Multiple imputation used m = 20 datasets generated via predictive mean matching (mice package), pooled using Rubin's (1987) rules with degrees of freedom estimated per parameter. All fifteen structural paths retained the same sign and a consistent significance pattern under multiple imputation as under the primary full information maximum likelihood (FIML) approach, and all indirect effects significant in the primary model remained significant under imputation. This confirms the primary findings are not an artifact of how missing predictor data were handled.

Table S11

Variable-Level Missingness

Variable	*N Total*	*n Valid*	*n Missing*	*% Missing*
Sport	801	801	0	0.0
Extracurricular	801	795	6	0.7
Exercise	801	736	65	8.1
Belonging	801	673	128	16.0
Loneliness	801	736	65	8.1
Stress Mindset	801	678	123	15.4
Flourishing	801	773	28	3.5
Depression	801	801	0	0.0
Anxiety	801	739	62	7.7
Gender	801	800	1	0.1
Age	801	801	0	0.0
Degree Level	801	798	3	0.4
Expatriate Status	801	781	20	2.5
Institution	801	801	0	0.0

Note. Of 801 participants, 733 (91.5%) provided complete data on all three engagement predictors and were retained in the primary path model; 68 participants (8.5%) had at least one missing predictor and were excluded, consistent with lavaan's default handling of exogenous variables under full information maximum likelihood.

Table S12

Comparison of Participants Included in and Excluded from the Primary Path Model

Variable	*M (Incl.)*	*SD (Incl.)*	*M (Excl.)*	*SD (Excl.)*	*t*	*p*
Flourishing	41.16	8.63	39.91	8.28	1.00	.324
Depression	12.45	6.86	6.41	7.75	6.20	<.001
Anxiety	10.22	6.05	11.24	6.07	-0.68	.504
Belonging	2.89	1.10	2.48	0.69	2.04	.064
Loneliness	6.16	1.91	6.29	1.89	-0.18	.864
Stress Mindset	10.06	2.65	11.4	2.50	-2.04	.060
Age	20.62	3.48	21.5	5.01	-1.42	.161
Gender (proportion female)	0.66	0.48	0.72	0.45	-1.13	.263
Expatriate (proportion)	0.56	0.50	0.53	0.50	0.38	.701

Note. Included n = 733; excluded n = 68. Participants excluded from the primary path model due to missing predictor data reported substantially and significantly lower depression scores than included participants, and marginally higher stress mindset and lower belonging. This indicates the missing-at-random assumption underlying full information maximum likelihood may not hold perfectly with respect to depression, and the analytic sample may modestly overrepresent students with elevated depressive symptoms. The multiple imputation sensitivity analysis reported in Table S10 addresses this concern directly.

Table S13

Distributional Properties and Screening Cut-off Proportions for PHQ-9 and GAD-7

Measure	*n*	*M*	*SD*	*Median*	*Min*	*Max*	*Skewness*	*Kurtosis*
Depression (PHQ-9)	801	11.94	7.14	11	0	36	0.40	-0.27
Anxiety (GAD-7)	739	10.24	6.04	10	0	21	0.17	-1.03

Proportions Above Established Screening Cut-offs

Cut-off	*Severity Level*	*n*	*%*
PHQ-9 ≥ 5	Mild	683	85.3
PHQ-9 ≥ 10	Moderate	473	59.1
PHQ-9 ≥ 15	Moderately Severe	278	34.7
PHQ-9 ≥ 20	Severe	121	15.1
GAD-7 ≥ 5	Mild	608	82.3
GAD-7 ≥ 10	Moderate	374	50.6
GAD-7 ≥ 15	Severe	200	27.1

Note. Skewness values within ±1 support the appropriateness of maximum likelihood estimation for these variables. Both instruments are validated screening tools rather than diagnostic instruments; proportions above established cut-offs indicate elevated symptom risk rather than confirmed clinical diagnoses.

Supplementary Code S1

Complete lavaan Model Syntax

Saturated Model

```
Belonging     ~ a1*SPORT + a2*EXTRAC + a3*EXERC
Loneliness    ~ b1*SPORT + b2*EXTRAC + b3*EXERC
StressMindset ~ c1*SPORT + c2*EXTRAC + c3*EXERC

Flourishing ~ d1*Belonging + d2*Loneliness + d3*StressMindset +
              cp1*SPORT + cp2*EXTRAC + cp3*EXERC
Depression  ~ e1*Belonging + e2*Loneliness + e3*StressMindset +
              cp4*SPORT + cp5*EXTRAC + cp6*EXERC
Anxiety     ~ f1*Belonging + f2*Loneliness + f3*StressMindset +
              cp7*SPORT + cp8*EXTRAC + cp9*EXERC

Flourishing ~~ Depression
Flourishing ~~ Anxiety
Depression  ~~ Anxiety
Belonging   ~~ Loneliness
Belonging   ~~ StressMindset
Loneliness  ~~ StressMindset
```

Trimmed Model

```
Belonging     ~ a2*EXTRAC
Loneliness    ~ b3*EXERC
StressMindset ~ c1*SPORT

Flourishing ~ d1*Belonging + d2*Loneliness + cp3*EXERC
Depression  ~ e1*Belonging + e2*Loneliness + e3*StressMindset +
              cp4*SPORT + cp6*EXERC
Anxiety     ~ f1*Belonging + f2*Loneliness + f3*StressMindset + cp9*EXERC

Flourishing ~~ Depression
Flourishing ~~ Anxiety
Depression  ~~ Anxiety
Belonging   ~~ Loneliness
Belonging   ~~ StressMindset
Loneliness  ~~ StressMindset

ind_ex_bel_flr := a2 * d1
ind_ex_bel_dep := a2 * e1
ind_ex_bel_anx := a2 * f1
```

```
ind_eb_lon_flr := b3 * d2
ind_eb_lon_dep := b3 * e2
ind_eb_lon_anx := b3 * f2
ind_sp_smm_dep := c1 * e3
ind_sp_smm_anx := c1 * f3
```

Both models were estimated using maximum likelihood with full information maximum likelihood (FIML) for missing data (lavaan version 0.6-12; Rosseel, 2012). Predictor covariances among the three exogenous engagement variables were freely estimated by default. Bootstrapped indirect effects reported in the main manuscript used 5,000 bias-corrected accelerated (BCa) resamples.
